# Primary Ovarian Pregnancy: A Case Report With a Review of the Literature

**DOI:** 10.7759/cureus.56688

**Published:** 2024-03-22

**Authors:** Sushma Bharti, Manupriya Sharma, Nisha Malik, Deychen Myes, Poojan Marwaha

**Affiliations:** 1 Pathology and Laboratory Medicine, All India Institute of Medical Sciences, Bilaspur, Bilaspur, IND; 2 Obstetrics and Gynaecology, All India Institute of Medical Sciences, Bilaspur, Bilaspur, IND

**Keywords:** emergency condition, maternal mortality, ovarian mass, ovarian pregnancy, ectopic pregnancy

## Abstract

Ectopic pregnancy (EP) constitutes 1%-2% of all pregnancies and is one of the leading causes of maternal morbidity and mortality. The most common site of ectopic pregnancy is the ampulla. Ectopic ovarian pregnancy (EOP) is one of the rare events, with an incidence of 0.5%-3% of all pregnancies. The incidence is higher in intrauterine device users or assisted reproductive techniques. The precise aetiology and pathogenesis of EOP remain elusive. Clinically, EOP mirrors the presentation of tubal pregnancy or a ruptured luteal cyst, often leading to life-threatening hypovolemic shock. Transvaginal sonography is the primary diagnostic tool. Still pinpointing the exact location early on poses challenges, and it’s usually misinterpreted as a tubo-ovarian mass, hemorrhagic cyst, or luteal cyst. Furthermore, while a suboptimal rise in serum beta-human chorionic gonadotropin (β-hCG) levels may indicate pregnancy, it doesn't definitively confirm EOP. Only histopathological examination offers a conclusive diagnosis. This paper discusses an EOP case in a young woman who experienced five months of amenorrhea and exhibited no traditional risk factors, underscoring the significant challenges inherent in preoperative diagnosis.

## Introduction

Ectopic pregnancy (EP) is one of the pregnancy complications where the embryo implants anywhere outside the uterus. The incidence of EP is about 0.91% in India [1] and 1%-2% in the United States [2]. The commonest site of EP is the ampulla, which constitutes 95% of all EPs. The non-tubal sites encompass the cervix, ovary, and abdomen, along with the hysterotomy scar due to a caesarean section or myomectomy. Rarely, a heterotopic gestation can be seen. The incidence of ovarian pregnancy (OP) among all EPs is 0.5%-3% in the United States [3]. It is a rare but well-known pathology. It is a commonly occurring gynaecological emergency and a leading cause of maternal mortality in the first trimester [1]. Herein, the authors report a case of a young pregnant female who presented in an emergency and was managed by ovarian wedge resection. This case contributes further to the literature on OP, emphasising the challenges faced during early preoperative diagnosis.

## Case presentation

**Figure 1:**
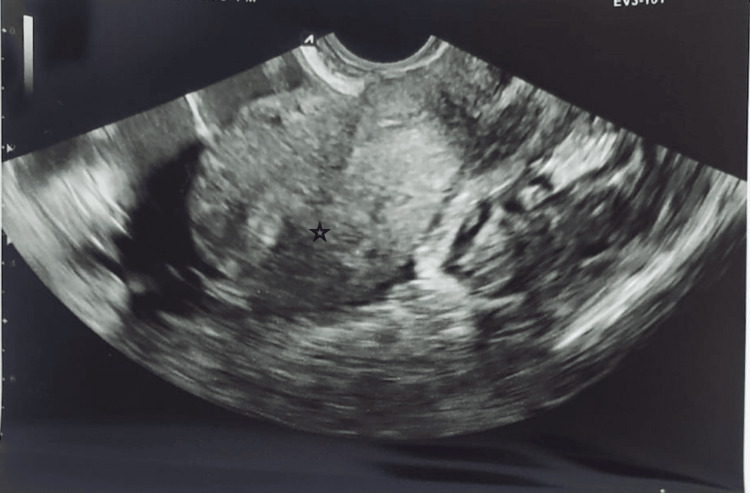
Ultrasonography shows an empty intrauterine sac (indicated by the star mark).

A 31-year-old female with a history of caesarean section presented with abdominal pain and bleeding per vagina for six to eight hours, with a history of five-week amenorrhea. There was no history of pelvic inflammatory disease (PID), sexually transmitted infections, or tuberculosis. She denied taking any oral contraceptive pills or intrauterine contraceptive devices (IUCD). Upon examination, there was mild pallor, a peripheral pulse of 120 per minute, a blood pressure of 100/60 mmHg, and lower abdomen tenderness. Per vagina, a normal-sized uterus, bilateral fornic tenderness with no palpable adnexal mass was noted. The urine pregnancy test was positive. Sonography did not show an intrauterine gestational sac (Figure 1).

**Figure 2:**
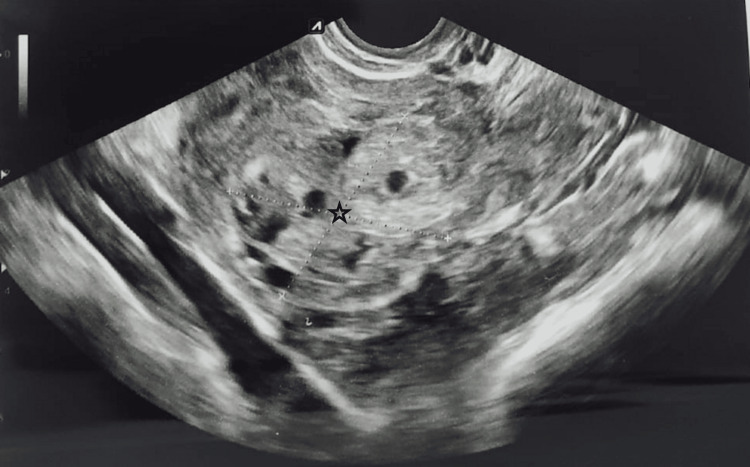
Ultrasonography shows increased vascularity and enlargement of the right adnexa (indicated by the star mark).

A heteroechoic right adnexal mass of 4.5 x 3.8 x 3.7 cm with increased vascularity was seen (Figure 2).

**Figure 3:**
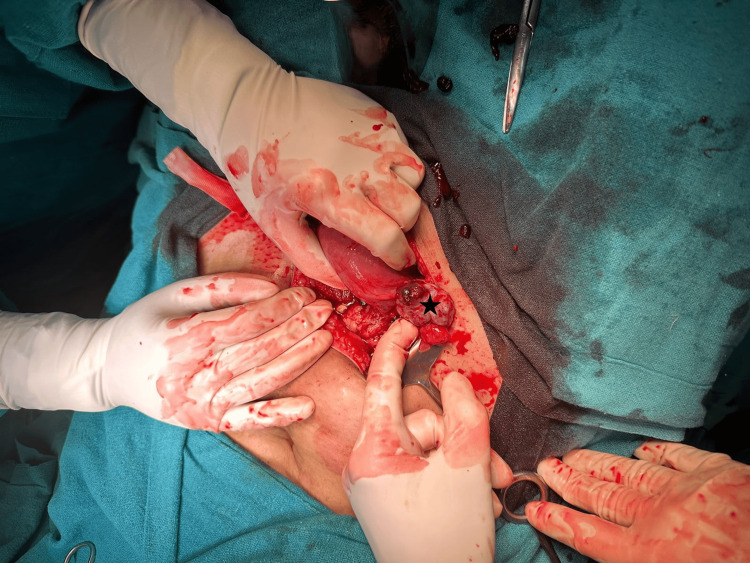
The intraoperative finding shows that the right ovary was enlarged with a bulging mass of 2x2 cm (indicated by the star mark).

With the clinical diagnosis of a ruptured EP/hemorrhagic cyst, an emergency laparotomy was planned. Intraoperatively, there was around one litre of hemoperitoneum. The right ovary was enlarged with a bulging mass of 2 x 2 cm and active bleeding from the surface (Figure 3). 

**Figure 4:**
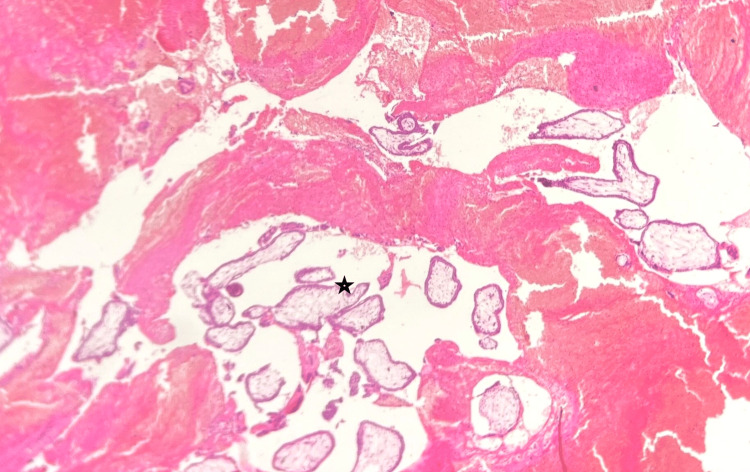
Microscopy shows chorionic villi and haemorrhage, as indicated by the star mark (40x, hematoxylin, and eosin stain).

**Figure 5:**
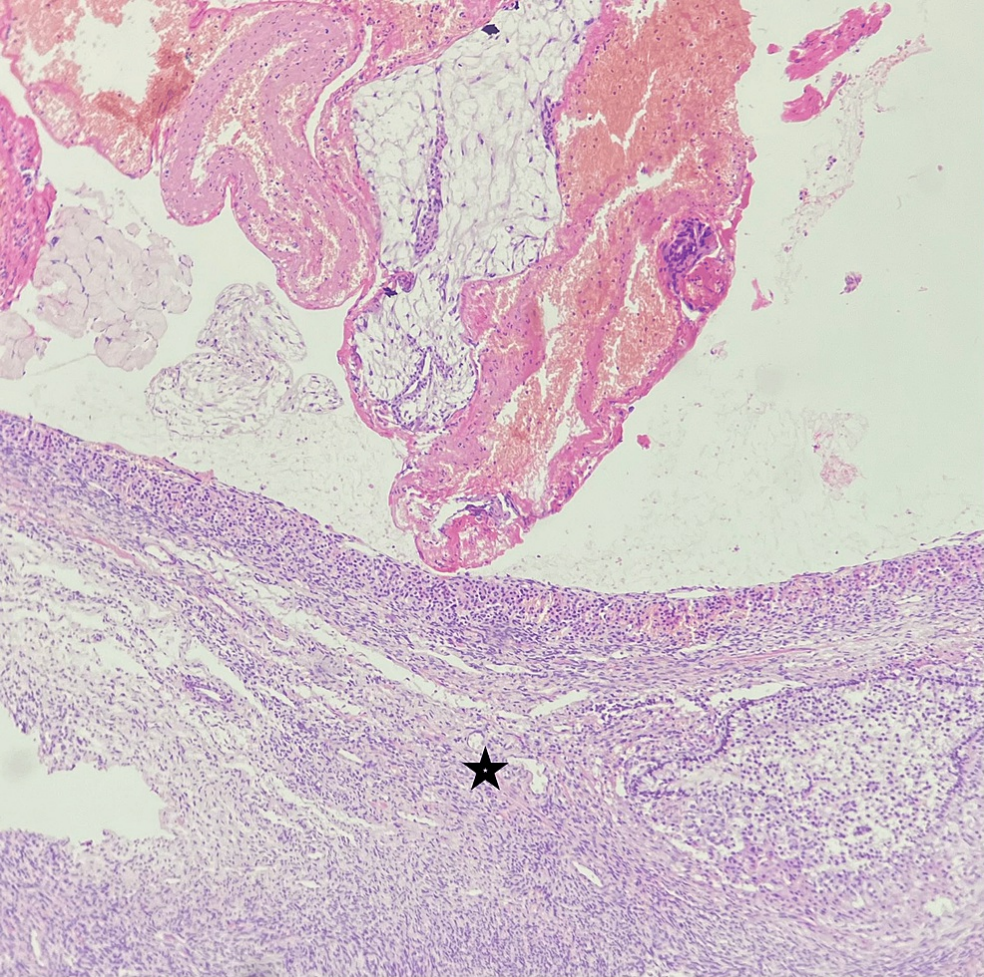
Microscopy shows ovarian parenchyma with blood clots inside, as indicated by the star mark (20x, hematoxylin, and eosin stain).

A wedge resection was done and sent for histopathology, which confirmed the presence of chorionic villi and decidua inside the ovarian parenchyma (Figures 4-5). 

The postoperative course was uneventful. The patient was discharged after one week in a stable condition.

## Discussion

An EP often emerges unexpectedly during the early stages of pregnancy. Frequently, women remain oblivious to its presence even after a positive urinary pregnancy test. The initial antenatal visit is crucial to ensuring the diagnosis of an intrauterine pregnancy (IUP), which traditionally occurs at two to six weeks. By this time, the gestation sac is established. However, first-trimester ultrasonography often misinterprets an OP as a tubo-ovarian mass. The first case of OP was reported by St. Maurice [4]. The incidence has surged since then, partly due to heightened awareness and the more widespread use of IUCDs and assisted reproductive technology (ART). Notably, between 57% and 90% of primary OP patients had used an IUD [5, 6].

In their research, Goyal et al. [3] discovered that 1.7% of all ectopic pregnancies and one in 3,332 deliveries were ovarian pregnancies. In a study by Chanu et al., the incidence of OP was as high as 6.3% [7]. Recent research also indicates that, if factors other than Speigelberg criteria are taken into account, the true incidence may be as high as one in 1,400 deliveries. The following are the Spielberg criteria for the confirmation of early ovarian pregnancy: a) the fallopian tube as the affected site must be intact; b) the foetal sac must occupy the position of the ovary; c) the ovary must be connected to the uterus by the ovarian ligament; and d) ovarian tissue must be located in the sac wall. [4]

The genesis of OP is attributed to the retention of a fertilised ovum within the ovary [8, 9]. An IUCD prevents IUP but not OP because it alters tubal motility [5]. Several risk factors predispose to tubal gestation, such as PID, endometriosis, and previous abdominal scarring. The risk factors for OP remain ambiguous. The data that suggest that patients having a caesarean section may have an increased risk of EP, especially scar ectopic, in subsequent gestation are limited. Yet, no evidence directly links caesarean procedures to OP. The use of IUD (19.3%), polycystic ovarian syndrome, in vitro fertilisation (IVF), and different fertility treatments (18.1%) is estimated to be the leading risk factor for OP [2]. Intriguingly, our patient’s history lacked these exposures. Also, no pelvis adhesions were found intraoperatively. Rarely, as in the present case, OP can occur without the presence of any classical risk factors.

An OP is usually detected within the seventh week of its initial scan. A surge in ovarian tissue vascularization frequently culminates in a rupture of 91% by the first trimester. Such ruptures lead to a medical emergency due to hypovolemic shock. Rupture in the second and third trimesters is rare and concerns 5.3% and 3.7% of cases, respectively [10]. Due to the delicate and non-elasticity of the ovarian cortex, ruptures frequently occur; finding intact gestational sacs on ovaries is a rarity, as observed in the current case.

Differentiating OP from conditions like ruptured luteal or hemorrhagic cysts can be challenging [11]. Non-visualisation of an IUP could indicate an extremely early IUP, a terminated pregnancy, or an EP, which can remain sonographically undetected. Also, its limited resolution may occasionally fail to delineate even advanced EPs, especially in non-compliant patients, or may reveal an adnexal mass that may not be classical for an EP. Additionally, distinguishing OP from conditions like missed abortions, tubal pregnancies, or hemorrhagic cysts preoperatively remains an intricate process, with a definitive diagnosis heavily reliant on histological examination [11]. Given fertility concerns, the predominant surgical approach involves laparoscopic wedge resection, preserving the undamaged ovarian tissue. Postoperatively, the beta-human chorionic gonadotropin (β-hCG) level gets normalised in about two weeks. Methotrexate is an alternative, but it has limited use because of the potential risk of massive bleeding and the consequent need for diagnostic laparoscopy [10]. Today, despite early diagnosis and treatment, which has significantly reduced morbidity and mortality, EP is still responsible for 10% of deaths in the first trimester. In our case, a salpingo-oophorectomy was deemed necessary after hemorrhagic infiltration and hematoma formation in the corresponding parametrium.

## Conclusions

An EOP remains a rare but significant medical challenge due to its deceptive clinical presentation and the inherent risks associated with its progression. Despite the myriad advancements in imaging techniques and diagnostic modalities, the definitive preoperative identification of EOP remains enigmatic. This particular case exemplifies the importance of high clinical suspicion in the face of atypical presentations and further highlights the integral role of histopathological examination in establishing a conclusive diagnosis. The case underscores the importance of prompt intervention, the efficacy of laparoscopic wedge resection, and the potential complications that can arise from overlooked or misdiagnosed conditions. Ensuring early detection and adequate treatment is imperative not only to safeguard the health of the mother but also to minimise complications that could compromise future fertility. Medical professionals should be acutely aware of the potential for EOP even in the absence of traditional risk factors, emphasising the unpredictability and uniqueness of each patient's clinical journey.
